# Unraveling the microbiome-metabolome nexus: a comprehensive study protocol for personalized management of Behçet’s disease using explainable artificial intelligence

**DOI:** 10.3389/fmicb.2024.1341152

**Published:** 2024-02-12

**Authors:** Sabina Tangaro, Giuseppe Lopalco, Daniele Sabella, Vincenzo Venerito, Pierfrancesco Novielli, Donato Romano, Alessia Di Gilio, Jolanda Palmisani, Gianluigi de Gennaro, Pasquale Filannino, Rosanna Latronico, Roberto Bellotti, Maria De Angelis, Florenzo Iannone

**Affiliations:** ^1^Dipartimento di Scienze del Suolo, della Pianta e degli Alimenti, Università degli Studi di Bari Aldo Moro, Bari, Italy; ^2^Istituto Nazionale di Fisica Nucleare, Sezione di Bari, Bari, Italy; ^3^Dipartimento di Medicina di Precisione e Rigenerativa e Area Jonica, Università degli Studi di Bari Aldo Moro, Bari, Italy; ^4^Dipartimento di Bioscienze, Biotecnologie e Ambiente, Università degli Studi di Bari Aldo Moro, Bari, Italy; ^5^Dipartimento Interateneo di Fisica ‘M. Merlin’, Università degli Studi di Bari Aldo Moro, Bari, Italy

**Keywords:** explainable artificial intelligence, microbiome, volatilome, Behçet’s disease, gut, butyrate, innate immunity, autoinflammatory disease

## Abstract

The presented study protocol outlines a comprehensive investigation into the interplay among the human microbiota, volatilome, and disease biomarkers, with a specific focus on Behçet’s disease (BD) using methods based on explainable artificial intelligence. The protocol is structured in three phases. During the initial three-month clinical study, participants will be divided into control and experimental groups. The experimental groups will receive a soluble fiber-based dietary supplement alongside standard therapy. Data collection will encompass oral and fecal microbiota, breath samples, clinical characteristics, laboratory parameters, and dietary habits. The subsequent biological data analysis will involve gas chromatography, mass spectrometry, and metagenetic analysis to examine the volatilome and microbiota composition of salivary and fecal samples. Additionally, chemical characterization of breath samples will be performed. The third phase introduces Explainable Artificial Intelligence (XAI) for the analysis of the collected data. This novel approach aims to evaluate eubiosis and dysbiosis conditions, identify markers associated with BD, dietary habits, and the supplement. Primary objectives include establishing correlations between microbiota, volatilome, phenotypic BD characteristics, and identifying patient groups with shared features. The study aims to identify taxonomic units and metabolic markers predicting clinical outcomes, assess the supplement’s impact, and investigate the relationship between dietary habits and patient outcomes. This protocol contributes to understanding the microbiome’s role in health and disease and pioneers an XAI-driven approach for personalized BD management. With 70 recruited BD patients, XAI algorithms will analyze multi-modal clinical data, potentially revolutionizing BD management and paving the way for improved patient outcomes.

## 1 Introduction

Human microbiome is the set of all the microorganisms that live in symbiosis with the human body, including bacteria, fungi, viruses and archaea. It has been found that, in a standard 70 kg male, bacteria are as numerous as somatic cells (Sender et al., 2016), but, due to their small dimensions, they contribute only 3% of the whole human body weight (Flint, 2012). Nevertheless, microbial communities are essential to keep the human body healthy. They synthesize some vitamins that our genes are not able to LeBlanc et al. (2013), help in the digestive processes (McConnell et al., 2008), teach the immune system how to recognize pathogens or cancer cells and even produce anti-inflammatory or anti-cancer compounds to defeat them (Nakkarach et al., 2021). The study of the human microbiome has demonstrated that microbial cell gene number in the human body are 150 times larger than our own genome (Zhu et al., 2010; Grice and Segre, 2012) and radically different collections of microbes have been found between different people. Scarce knowledge about what are the causes of these variations and what regulates them has been achieved. A very impactful issue is that no understanding on how the human microbiome modification has influence on wellness, conservation of health, starting and rise of diseases has been reached (Gilbert et al., 2018; Mandrioli et al., 2019). However, a correlation between changes in the microbiome, its metabolome and interaction with the immune, endocrine and nervous systems and the appearance of a wide spectrum of diseases [e.g., inflammatory bowel disease (Frank et al., 2007; Gevers et al., 2014; Ni et al., 2017), cancer (Kostic et al., 2013) or depressive disorders (Jiang et al., 2015; Zheng et al., 2016)] has been detected. This finding indicates the possibility of treating this kind of illness by manipulation of such a microbial community. Variations in human oral or intestinal microbiome and its volatilome can mirror host lifestyle and affect the levels of diseases biomarkers (Vernocchi et al., 2020). The comprehension of the relationships between host microbiome and phenotypes is of fundamental importance to understand health or disease states. Similarly, chemical characterization of human breath and the identification of volatile organic compounds (VOCs) patterns linked to a specific disease, can provide information on the health state of a patient and allow early diagnosis of chronic diseases or the monitoring of the patient’s health state along therapeutic follow-up. In fact, VOCs are final products of cellular metabolic processes and their nature and/or concentration in human breath change along with metabolic pathways when a pathologic state onsets (Mozdiak et al., 2019).

Data from human microbiome and breath are inherently complex, noisy and highly variable because several factors such as diet, sex, hormonal status, drugs, habits, etc. could affect them. So, non-standard analytical methodologies are needed to extract their clinical and scientific potential. Nowadays, a lot of Artificial Intelligence (AI) methods, such as Machine Learning (ML) or complex networks, are available to catch this complexity. In particular, AI methods use several layers of linear and/or non-linear calculating units to understand the data they manipulate and to learn “patterns” from the same data. This learning can be used to classify the observations or to make predictions on them (Hassabis et al., 2017; Amodeo et al., 2021). The specific AI model to be used is chosen according to its capability to maximize prediction accuracy but requires, on the other hand, an increased complexity of the model itself, that makes it less interpretable (Shaban-Nejad et al., 2021) (e.g., “black boxes”). To overcome these drawbacks, coming from more complex models, and to adapt ML utilization to clinical contexts, eXplainable Artificial Intelligence (XAI) techniques have been introduced, that provide explanations for decisions the algorithm takes and for the risk scores calculated for every subject studied. Such a gain in interpretability for the chosen model is converted in the possibility to understand the main reasons standing behind a prediction and to point out the factors that majorly affect clinical risk scores at individual level. This approach is perfectly placed in an innovative concept of Personalized Medicine that requires the help of AI techniques.

The target of the proposed study is the Behçet Disease (BD), also known as Silk Road disease, a rare, complex and multi-systemic chronic vasculitis, characterized by mucocutaneous, articular, vascular and ocular lesions and also by central nervous system (CNS) symptoms. The most recurring signs of this disease are relapsing genital and oral aphthae (that can also spread in the whole digestive tract), ocular pathologies (>50% of cases), arthralgia and/or arthritis (45% of cases), venous system vasculitis and thrombosis. If thromboses occur in the arterial system, they usually involve pulmonary vessels. Neurological signs (neuro-BD) are frequent (>20%); they often occur 1–10 years after the first symptoms, and include headache, hemiparesis, behavior alterations and sphincter dysfunctions. Nowadays, BD etiology is still not clear and cannot be traced back to a single root cause: the overactivation of the innate immune system, typical of this disease, seems to be caused by an altered T-cells homeostasis, but it is common thought that also some components of the human microbiome can promote an abnormal adaptive immune response, in presence of a favorable genetic background (Rodrìguez-Carrio et al., 2021). In fact, several studies have linked BD to an intestinal or oral microbiota dysbiosis: in particular, a decrease in number of butyrate-producing bacteria, associated to a lower level of butyrate in fecal samples of patients has been noted (Consolandi et al., 2015). As concerning gut, butyrate is involved in regulatory T cells differentiation (Furusawa et al., 2013) and in the release inhibition of pro-inflammatory cytokines (Weng et al., 2007). Low production of butyrate in patients suffering from BD may cause both reduced T-reg responses and T-cells immune-pathological responses activation, as suggested by the prevalence of T helper cells Th1 and Th17 in patients affected by BD (Alpsoy, 2016). Influencing intestinal microbiota, with factors such as the diet, can have a role in correcting intestinal dysbiosis and in reducing the severity of BD active phases. The evidence collected in the last decade highlight that adhering to dietary patterns which include high content of fibers can be linked to a better intestinal microbiota equilibrium; such a condition is favorable for short chain fatty acid (SCFA) producer bacteria and unfavorable for bacteria species associated to a pro-inflammatory pattern (Fu et al., 2020). Microbiota associated with dietary patterns rich in fibers was found to be positively correlated with high levels of SCFA (acetate, propionate, butyrate). Intestinal microbiota produces SCFA during indigestible polysaccharides (fibers) fermentation; these acid compounds have a well-documented protective role against several pathologies (Ho et al., 2018). To the best of our knowledge, a well-defined diet plan for BD does not exist, and the general advice is to follow a balanced diet and to maintain an ideal weight. Nevertheless, the just mentioned studies allow us to speculate that following a diet rich in fiber can correct intestinal dysbiosis, which is involved in the BD pathogenetic mechanism, and stimulate butyrate endogenous production from intestinal microbiota, bringing to a potential improvement of clinical manifestations.

Keeping all these evidence in mind, the proposed study is aimed to: (i) establish correlations between oral and intestinal microbiota, fecal and salivary volatilome, breath and phenotypic features of human hosts, affected by BD, active and/or in remission; (ii) identify, through cluster analysis methods of metabolites, different groups of patients affected by BD; (iii) identify some taxonomic units of oral and fecal microbiota and metabolic markers that majorly contribute to the prediction of different clinical outcomes (e.g., number of active mucosal lesions, remission following the Behçet Disease Current Activity Form (BDCAF)); (iv) identify, with XAI methods (Bellantuono et al., 2022), some personalized metabolic markers that, for each patient, contribute to the prediction of his/her clinical outcome (personalized medicine); (v) evaluate the effects of soluble fiber intake (inulin) on eubiosis/dysbiosis conditions of oral and intestinal microbiota and on endogenous production of butyrate; and (vi) establish correlations between eating habits and clinical outcome of patients.

## 2 Methods and analysis

### 2.1 Study design

The project we are going to propose will be performed in three different sub-activities. The first sub-activity includes a two-arm randomized study (duration: 3 months): patients in the control arm will keep on assuming the standard therapy while patients in the treatment arm will assume soluble inulin-type fructans (inulin 90% from *Cichorium intybus L.*; Farmalabor S.r.l., Canosa di Puglia, Italy), along with the standard therapy. At the starting of study and 3 months later, for each patient, the following samples and data will be collected:

- samples for the assessment of oral/fecal microbiota;
- breath samples;
- clinical data such as Body Mass Index (BMI), disease duration, clinical phenotype and ocular, articular or mucocutaneous involvement;
- laboratory data such as Erythrocyte Sedimentation Rate (ESR) and C-reactive protein (CRP);
- information on breath components;
- information about eating habits, inviting patients to keep a food diary that can provide detailed descriptions on type and quantity of food and beverages consumed.

Furthermore, the second sub-activity will consist of:

- analysis of volatilome in breath samples and microbiota in saliva and fecal samples;
- analysis of bacterial community taxonomic composition in fecal and saliva samples;
- chemical characterization of breath samples.

Volatile metabolites (volatilome) chemical characterization in breath samples will be determined through gas-chromatography coupled with mass spectrometry (GC–MS). For quality assurance in sampling phase and avoid any environmental contamination of breath samples, the end-tidal fraction of the exhaled breath will be collected by an automated device named Mistral (Predict srl) and directly transfer onto suitable adsorbent cartridges (Bio-monitoring steel tube, Markes International Ltd., UK) that will be preconditioned at 330°C for 30 min with pure helium (99.999%), analyzed to verify VOCs background level and properly stored at 4°C until use. Once collected onto the adsorbent cartridges, VOCs will thermally desorb and analyze by means a thermal desorber (UNITY-2, Markes International Ltd.) coupled with a gas chromatograph (GC 7890, Agilent Technologies) and a mass selective detector (MS 5975, Agilent Technologies). The analytical methodology for VOCs characterization in breath samples has been already optimized and validated in previously published studies (Di Gilio et al., 2020a,b). With the purpose to emphasize the chemical information related to human metabolomics and identify the most part of endogenous VOCs of interest (not exclusively those included in standard mix) a semi-quantitative analysis based on compound abundances will be performed. More specifically, the GC–MS chromatograms will be analyzed using the GC–MS post-run analysis software (Agilent Mass Hunter Qualitative Analysis-Agilent Technologies Ltd., Santa Clara, USA) integrating only the peaks with intensity higher than 5 times than baseline and VOCs compounds will be identified through spectral library matching (Compounds library of the National Institute of Standards and Technology, Gaithersburg, MD 20899–1070, USA) and through comparison with GC–MS chromatograms obtained by analysis of standard solutions of 44 VOCs (Ultra Scientific Cus-5997). Microbiota composition study will be performed through metagenetic analysis of rRNA16S gene (V3 and V4 regions). A negative control for sequencing will be included in the workflow of 16S amplification and library preparation, consisting of all the reagents included in the sample processing and without the sample, to ensure that no contamination took place. Libraries will be quantified using a Qubit fluorometer (Invitrogen Co., Carlsbad, CA, USA) and pooled, including the Phix control library, to an equimolar amount (4 nM final concentration). FastQ file quality will be assessed by using FastQC software and analyzed by using the QIIME2 dedicated pipeline^1^ microbiome platform (version 2020.8). Denoising will be computed with the q2-deblur QIIME plugin. Taxonomy will be inferred with the QIIME-compatible database Silva v.138 SSU, using an amplicon sequence variant (ASV) table based on error-corrected reads (Calabrese et al., 2022; Vacca et al., 2022, 2023). Finally, the last sub-activity is devoted to the implementation of XAI methods: the data obtained with the previous sub-activities will be analyzed with innovative AI methods. The aim will be to evaluate the conditions of eubiosis/dysbiosis and to identify potential microbial and metabolic markers linked to BD, to eating habits of patients and to a soluble fiber dietary supplement administration. The estimated project duration should be 18 months, including the enrollment time.

### 2.2 Study population

The study will be conducted on patients with BD, active or in remission according to BCDAF, aged from 18 to 65, after having signed the informed consent for participating in the study and for assuming inulin. Exclusion criteria will include pregnancy and breastfeeding, serious concomitant diseases or instability conditions (such as autoimmune diseases, chronic viral infections, malignant cancers), recent myocardial infarction (MI), chronic liver diseases and inflammatory bowel diseases (IBD) and recent (last 6 months) or current participation to slimming programs or assumption of weight loss drugs.

### 2.3 Interventional method

The fiber dietary supplement will be administered randomly to half of the study patients, in open-label mode. The BD patients will receive either inulin supplementation or placebo. The participants were recommended to consume the powder during the breakfast by mixing it to 150 mL of warm water and then stirring up the powder until dissolved.

At the starting point and 3 months later, for each BD patients will be collected: samples for the assessment of oral/fecal microbiota, breath samples, BMI, disease duration, clinical phenotype and ocular, articular or mucocutaneous involvement and information about eating habits. Patients in the treatment arm will assume 5 g per day of inulin in addition to their ordinary diet and in a randomized order. The 5 g dose was chosen after considering the amounts of prebiotics that would be sufficient to induce positive and significant changes in the gut microbiota, but low enough to avoid adverse effects and minimize gastrointestinal discomfort (Bouhnik et al., 1999; Kolida et al., 2007).

All data obtained, will be analyzed with innovative AI methods, in order to evaluate the conditions of eubiosis/dysbiosis and to identify potential microbial and metabolic markers linked to BD, to eating habits of patients and to a soluble fiber dietary supplement administration.

### 2.4 Sample size estimation

To evaluate the differences, in terms of beta-diversity, in the whole microbial population, calculating the mean presence of operative taxonomic units (OTUs) between two groups with *α* = 0.05, 1-β = 0.80, final effect size = 0.80, the enrollment of 26 patients is needed. Taking into account a 20% dropout rate, an amount of 35 patients for each group is needed, with a total number of 70 patients for the whole study. For the univariate logistic regression with significance level 1-*β* = 0.80 and *α* = 0.05, the target is to detect a shift of the probability (P_0_) (*Y* = 1) from the value of 0.10 regarding the mean value of X to the value of 0.30 when X is increased by a standard deviation above its mean value. This outcome corresponds to an Odd Ratio (OR) of about 3.80, which requires a total sample size of 90 patients to provide a two-tail significance test. In the end, a total of 70 patients has been taken into account as the minimum number necessary for the study, because it will be needed to implement multivariate models for the adjustments. In fact, considering an expected squared multiple correlation coefficient between the covariates of about 0.30, to be included into the multivariate models, the minimum sample dimension increases to 70 patients for the two-tail significance test. Finally, a group of 70 patients with BD, classified according to ISG and/or ICBD criteria, will be selected for this study. The features of this cohort are the following: 15/70 patients with mucocutaneous involvement (active or in remission, according to Behçet’s Disease Current Activity Form criteria) and 55/70 patients with articular involvement (active or in remission).

### 2.5 Outcome measure

In the initial phase of our study, our primary focus lies in a data-driven analysis designed to distinguish, at the 3-month period, two distinct patient groups based on microbiota and volatilome profiles. The first group undergoes traditional treatment with soluble fiber intake (inulin), while the other receives only traditional treatment. This outcome is propelled by the application of Explainable Artificial Intelligence (XAI) techniques, aiming to uncover the pivotal features contributing to the differentiation between the two groups. Our investigation extends to understanding the global and local importance of these features, providing insights into the personalized metabolic responses to treatment.

The outcome measures considered are summarized in Table 1. Integrating these biological and clinical parameters using a data-driven approach, our objective is to paint a comprehensive picture of the personalized metabolic markers associated with Behçet’s disease. This dual-phase evaluation not only enriches our understanding of microbiome and metabolome nexus with the disease but also lays the groundwork for targeted interventions and more detailed treatment strategies.

**Table 1:** Biological and clinical outcome measures considered in the presented protocol study.

Biological outcome measures	Erythrocyte sedimentation rate (ESR)
	C-reactive protein (CRP)
Clinical outcome measures	Behçet’s Disease Current Activity Form (BDCAF) – measure of disease activity
	Krause Total Severity Score – measure of disease severity
	Short-form (SF)-36 quality of life (QoL) scale – measure of disease QoL

### 2.6 Adverse events

Symptoms relating to gastrointestinal discomfort (abdominal discomfort, diarrhea, constipation, bloating, and flatulence) are widely reported in human prebiotic feeding studies, but they remain very mild at recommended intakes (Rumessen et al., 1990; Gibson et al., 1995). Based on the literature, 16 g of inulin-type fructans per day induces no or only minor gastrointestinal symptoms in healthy or diseased adults (Cani et al., 2009; Birkeland et al., 2020). Taking potential side effects into consideration, 5 g dose was preferred over higher doses due to a precautionary principle.

### 2.7 Data recording and data monitoring

Follow-up assessments and data collection will be undertaken at the U.O.C. Reumatologia Universitaria of the Policlinico Hospital, Bari, Italy, by trial personnel.

### 2.8 Data analysis

Data collected by investigators will include volatilome, oral/fecal microbiota, body mass index (BMI), disease duration, clinical phenotype and ocular, articular or mucocutaneous involvement.

The microbiota can be characterized in three different ways: alpha diversity metrics, relative abundance of phylotypes for each specimen and community state types (CST). Alpha diversity metrics, which represent the variety and richness of organisms in a specimen, and relative abundance of microbes will be analyzed through supervised machine learning algorithms as Random Forest or XGBoost classifiers. Supervised machine learning is a category of machine learning where the algorithm is trained on a labeled dataset, which means that each example in the training data is associated with the correct output or target. The algorithm learns to make predictions or decisions based on input data by generalizing from the labeled examples it has seen during training. Moreover, the XAI algorithm “SHapley Additive exPlanations” (SHAP) will be used to detect for each patient, which features are more important for the ML algorithm in its classification (Bellantuono et al., 2023; Novielli et al., 2023). SHAP is an algorithm used in machine learning to explain the predictions made by complex models, particularly for models like XGBoost, Random Forest, neural networks, and others. It provides interpretable explanations for individual predictions, helping users understand why a particular prediction was made. The third characterization, i.e., CST, which groups samples according to the composition of the microbiota, will be analyzed through the application of complex networks (CN). This mathematical method, also known as complex systems or complex networks theory, is a branch of network science that studies systems characterized by a large number of interconnected components or nodes, and the patterns and properties that emerge from these connections. In our case, interactions between microbiome and its host are complex phenomena, and to better understand this kind of complex interactions and to map microbiome behavior is of fundamental importance to have the possibility to model these interactions through CN. Modules of this complex biological network are key organizational elements for the network itself. To detect modular organizational structures of a complex network, community detection unsupervised algorithms will be used.

### 2.9 Comprehensive methodology for data challenges

To ensure a robust evaluation of our models, we will implement a cross-validation strategy. Cross-validation involves partitioning the dataset into subsets, training the model on some of these subsets, and testing it on the remaining subset. This process will be repeated multiple times, and the performance metrics will be averaged. This approach ensures that our models generalize well and helps prevent overfitting.

To handle the possible presence of missing values, we will adopt a two-fold approach:

1. *Variable Selection*: Variables with a relatively low percentage of missing values (below a defined threshold, e.g., 30%) will be considered to maintain data quality.
2. *Imputation Techniques*: For variables exceeding the threshold, established imputation techniques will be employed. Additionally, we will use imputation methods such as replacing missing values with the mean or maximum of the respective variable. Importantly, these techniques will be applied separately to the training and testing datasets to prevent data leakage and ensure model generalization to unseen data.

We would also like to highlight the utility of the XGBoost algorithm, which inherently handles missing values in tree algorithms by learning branch directions during training.

To handle the potential limitation in the number of available patterns compared to the number of features considered, which could lead to overfitting, we will address the issue through the implementation of two robust techniques: data augmentation and feature reduction.

1. *Data Augmentation*: The data augmentation strategy aims to artificially amplify the quantity of training samples for deep learning models, emulating the distribution of the original dataset. This becomes especially advantageous when confronted with the constraint of a limited size in the training dataset. By introducing more diverse instances, it facilitates the model in generalizing more effectively, tackling the challenge posed by smaller training datasets. Essentially, it functions as a preprocessing technique and a type of regularization, significantly enhancing model performance and mitigating the risk of overfitting. Furthermore, the integration of Generative Adversarial Networks (GANs) into data augmentation further expands its capabilities. GANs can be employed to simulate data, generating synthetic instances that closely resemble real data. This innovative use of GANs not only augments the dataset but also introduces a layer of complexity and realism, ultimately contributing to the model’s ability to generalize and perform effectively across diverse scenarios (Creswell et al., 2018).
2. *Feature Reduction*: Feature reduction is a crucial aspect of our approach. Techniques such as Principal Component Analysis (PCA) (Song et al., 2010) and wrapper methods like Boruta (Kursa et al., 2010; Bellantuono et al., 2023) will be employed. These methods effectively reduce the dimensionality of the feature space, allowing us to train models even with a limited number of instances. This not only aids in computational efficiency but also contributes to model interpretability.

### 2.10 Ethics approval

This study has been approved by Comitato Etico Indipendente, Azienda Ospedaliero-Universitaria ‘Consorziale Policlinico’ on February 2023 (prot. n. 0023249|09/03/2023).

## 3 Discussion

### 3.1 Choice of treatment

Behçet’s disease is a rare, chronic, autoimmune disorder that can affect blood vessels throughout the body. It is named after the Turkish dermatologist, Hulusi Behçet, who first described the condition in 1937. This disease primarily involves inflammation of blood vessels (vasculitis) and can affect various parts of the body. The overactivation of the innate immune system, typical of this disease, seems to be caused by an altered T-cells homeostasis, but it is common thought that also some components of the human microbiome can promote an abnormal adaptive immune response, in presence of a favorable genetic background. Behçet’s disease is more common in certain regions, such as the Mediterranean, Middle East, and Asia, but it can affect people of any ethnicity. Diagnosis is often based on clinical symptoms and may require ruling out other similar conditions. Treatment typically focuses on managing symptoms and reducing inflammation.

The gut microbiome has been a subject of extensive research in the context of immunological diseases. A recent study showed that a peculiar dysbiosis of the GM is present also in individuals with BS, mainly represented by a depletion of SCFA-producing bacteria, especially of butyrate (Pagliai et al., 2020). Several trials previously showed that inulin-type fructans supplemented in doses varying between 5 and 30 g per day may increase the SCFA levels and enrich microbial diversity in healthy and diseased people (Gibson et al., 1995; Ramirez-Farias et al., 2008; Calabrese et al., 2022; Vacca et al., 2023). Thus, the aim of the present project is to conduct a trial to investigate whether a supplement of inulin could be beneficial for the gut microbiome and metabolome to the amelioration of the clinical symptoms and disease severity in individuals with BS. In support, a previous proof-of-concept study demonstrated that butyrate-enriched diets modulate the redox state of the blood and promote fibrin degradation, which is impaired by a neutrophil-dependent mechanism in BS (Becatti et al., 2016). However, the same study reported no significant effects on gut microbiota composition and SCFA production, suggesting that more effective dietary interventions are needed (Emmi et al., 2021).

### 3.2 Anticipated results

This will be the first study that tries to understand the complex relationships between diet, intestinal microbiota and human breath in patients affected by BD through an innovative approach based on AI methods (Golob et al., 2023; Novielli et al., 2023; Papoutsoglou et al., 2023). Such an understanding can represent a significant step forward toward the comprehension of pathogenetic mechanism at the basis of BD onset and the identification of microbial, metabolic and immunological factors and therapeutic biomarkers able to control treatment outcome and to better understand how the such a treatment can modify microbiome. In fact, intestinal dysbiosis has been linked to inflammatory diseases (Douzandeh-Mobarrez and Kariminik, 2019) and recent studies have demonstrated that therapeutic treatment in rare rheumatological diseases can modify subclinical intestinal inflammation and dysbiosis (Manasson et al., 2020), highlighting the bidirectional nature of this correspondence. Furthermore, this study will evaluate for the first time with multivariate models if microbiome and breath modulation through the diet can improve disease activity in patients with BD under treatment. This analysis could enable us to find valuable markers to identify responders and non responders, allowing treatment optimization and a personalized therapeutic approach. This study could be also useful to analyze diet effects on BD activation and/or remission. Going into details, network approach thought for this study is aimed to catch functional structure of dynamic processes happening between microbiome and human host, to identify the coexistence of different microorganisms, to trace relationships between microorganisms and to identify cohesive groups that play fundamental roles in maintaining functional relationships in the global network during the treatment. Identification and quantification of some of the topological properties of the network modules can provide important information on microbiome interactions and on their relationship with possible disorders and anomalies in inflammatory and pathological states. Specifically, co-occurrence patterns and identified polymicrobial interactions will be related with other clinical and phenotypical data to detect correlations between network functional and structural properties and biological and pathological profiles in different starting conditions. This integrative approach is completely innovative, since it will allow to highlight some connectivity patterns linked to inflammatory states, pathologies, etiological agents and even the organisms responsible for pathology transmission.

In our study protocol, we propose groundbreaking methodologies for personalized understanding of Behçet’s disease. One avenue of exploration involves the utilization of breath analysis to identify distinct Volatile Organic Compounds (VOCs) patterns in exhaled breath (Di Gilio et al., 2020a). By harnessing the capabilities of artificial intelligence algorithms, we aim to explore the nexus between microbiome and metabolome offering a non-invasive and efficient approach for Behçet’s disease management. Here, machine learning takes center stage, enabling us to unravel complex patterns within the oral microbiome. The goal is to uncover unique microbiome signatures associated with Behçet’s disease, laying the groundwork for a personalized medicine approach. This exploration promises not only a deeper understanding of the disease but also the potential for tailored interventions based on individualized oral microbiome and metabolome profiles (Bellando-Randone et al., 2021).

In the third facet of our study, we introduce the application of explainable artificial intelligence to analyze microbiome and volatilome data related. This innovative approach addresses the limitations of traditional machine learning methods, offering a clear and interpretable understanding of disease-associated microbiome and metabolome biomarkers. By incorporating local explanation embeddings and an unsupervised clustering method, we could anticipate the identification of distinct subgroups among subjects (Novielli et al., 2023). These perspectives open the door to personalized interventions, marking a significant stride toward a more nuanced and effective treatment paradigm for Behçet’s disease.

## 4 Conclusion

The protocol presents a promising and innovative approach to understanding BD, with potential implications for personalized treatment strategies, using eXplainable Artificial Intelligence.

The versatility of the selected analysis methods makes it possible to apply this approach to other types of complex diseases.

## Data availability statement

The original contributions presented in the study are included in the article/supplementary material, further inquiries can be directed to the corresponding author.

## Author contributions

ST: Conceptualization, Funding acquisition, Methodology, Project administration, Supervision, Writing – original draft, Writing – review & editing. GL: Conceptualization, Methodology, Supervision, Writing – review & editing. DS: Methodology, Writing – review & editing. VV: Methodology, Writing – review & editing. PN: Methodology, Writing – original draft, Writing – review & editing. DR: Methodology, Writing – review & editing. AG: Methodology, Writing – review & editing. JP: Methodology, Writing – review & editing. GG: Methodology, Supervision, Writing – review & editing. PF: Conceptualization, Methodology, Writing – review & editing. RL: Writing – review & editing. RB: Methodology, Writing – review & editing. MA: Conceptualization, Methodology, Supervision, Writing – review & editing. FI: Conceptualization, Funding acquisition, Methodology, Supervision, Writing – review & editing.

## Footnotes

^1^https://qiime2.org
